# Enhanced catalytic efficiency of CotA-laccase by DNA shuffling

**DOI:** 10.1080/21655979.2019.1621134

**Published:** 2019-05-29

**Authors:** Fengju Ouyang, Min Zhao

**Affiliations:** a Institute of advanced technology, Heilongjiang Academy of science, Harbin, China; b Department of Microbiology, Northeast Forestry University, Harbin, China

**Keywords:** *Bacillus* species, CotA-laccase, DNA shuffling, Dyes decolorization

## Abstract

Bacterial CotA-laccases exhibit higher activity in alkaline pH and salt concentration conditions compared to laccases from white-rot fungi. They are considered as green catalysts in decolorizing of industrial dyes. However, CotA-laccases are limited due to the low yield and catalytic efficiency as the spore-bound nature of CotA. A DNA shuffling strategy was applied to generate a random mutation library. To improve laccase activities, a mutant (T232P/Q367R 5E29) with two amino acid substitutions was identified. The catalytic efficiency of mutant 5E29 was 1.21 fold higher compared with that of the wild-type. The Km and k_cat_ values of 5E29 for SGZ were of 20.3 ± 1.3 µM and 7.6 ± 2.7 s^−1^. The thermal stability was a slight enhancement. Indigo Carmine and Congo red were efficiently decolorized by using this mutant at pH 9.0. These results provide that 5E29 CotA-laccase is a good candidate for biotechnology applications under alkaline condition, with an effective decolorization capability.

## Introduction

Laccases (benzenediol: oxygen oxidoreductase, EC 1.10.3.2) belong to the multi-copper oxidase family and catalyze the reduction of molecular oxygen to water by the oxidation of substrate molecules, such as polyphenols, polyamines and certain inorganic ions[1]. Laccases are widely distributed in nature, including higher plants, fungi, bacteria and insect [2]. Industrial dye wastewaters are always characterized at non-natural conditions, e.g. extreme pH values (8.0–11.0) and high temperature, which leads to the inactivation of fungal laccases [3]. Spore coat protein A (CotA) from *Bacillus subtilis*, a 65 kDa protein (513 amino acids), is a high thermostable bacterial laccase [4]. The CotA-laccase has been reported for industrial textile wastewater detoxification and decolorization [5,6]. Low production yield of the native CotA-laccase limited the applications in industry. The researches have focused on the production of CotA laccase by genetic engineering. *Escherichia coli* is considered the most preferable recombinant protein expression system due to its rapid growth and easy genetic manipulation and considered as the most dynamic system for industrial biocatalysts [7]. The CotA-laccases orthologs from different *Bacillus* species such as *B.subtiils* [8], *B.pumilus* [9], *B.amyloliquefaciens* [4], *B.licheniformis* [10], *Bacillus clausii* [11], *Bacillus vallismortis* [12] and *Bacillus sphaericus* [13], have been expressed heterologously in *E. coli*. However, this expression pattern often results in inactive inclusion bodies and low catalytic effiency. These shortcomings should be resolved through directed evolution. Directed molecular evolution is an extremely powerful approach to tailor enzymes for particular purposes of application by mimicking in the lab the key processes of natural evolution. CotA-laccases have the predictable robustness for the destabilizing conformational changes without disturbing function, so they are particularly suited for a molecular-directed evolution strategy [14].

The objective of the current study was to generate evolved recombinant proteins with improved catalytic activities. We developed a novel DNA shuffling method in order to construct a random mutagenesis library in recombinant *E. coli* strain. A mutant, 5E29 was identified and characterized to be a 1.21-fold higher catalytic activity. The biochemical properties of the wild-type and variant CotA-laccase were compared. Moreover, the dye decolorization capacity of this mutant was evaluated.

## Materials and methods

### Materials

2,2′-Azino-bis (3-ethylbenzothiazoline-6-sulfonic acid; ABTS), syringaldazine (SGZ), Isopropyl-β-D-thiogalactopyranoside (IPTG), acetosyringone (ACS) and Indigo Carmine were purchased from Sigma-Aldrich (St. Louis, MO, USA). Universal DNA Purification Kit was purchased from Tiangen (Beijing, China). BugBuster Protein Extraction Reagent was purchased from Novagen (Merck, Billerica, MA, USA). All chemicals were of analytical grade or higher.

### Strains, media, and plasmids

The CotA-laccase genes from *B. subtilis* LS03 and *B. amyloliquefaciens* LS05 strain isolated by our laboratory were expressed into pETDuet-1 supplied by Invitrogen (Carlsbad, CA). The pMD18-T plasmid, T4 DNA Ligase and restriction enzymes were purchased from Takara (Dalian, China). Luria-Bertani (LB) medium was used to cultivate all strains.

### Bioinformatics analysis

The modeling was carried out by submitting the deduced protein sequence of CotA-laccase to the Swiss Model server (https://swissmodel.expasy.org/). The *B. subtilis* LS03 shared 99.2% sequence identity with the modeling one (PDB code: 1GSK). Hydrogen bonds of the wild-type CotA and its variant were investigated using SPDBV software version 4.10 and PIC (http://pic.mbu.iisc.ernet.in/).

### Library creation

CotA laccase gene of *B. subtilis* LS03 and *B. amyloliquefaciens* LS05 were isolated by following the instructions of ‘Universal DNA Purification Kit’. These CotA genes were amplified by PCR using two pair primers in Table 1.

**Table 1.** Sequences of specific primer pairs used for cloning procedures.

Genes	Primer name	Primer sequences (from 5ʹto 3ʹ)
*B. subtilis* LS03	Forward (CotA-3F)	CGGGGATCCGACACTTGAAAAATTTG
	Reverse (CotA-3R)	CCGCTCGAGTTTATGGGGATCAG
*B. amyloliquefaciens* LS05	Forward (CotA-5F)	CGGGAATTCGGCACTGGAAAAATTTGCAGATG
	Reverse (CotA-5R)	CCGCTCGAGCTGCTTATCCGTGACGTCCAT

DNA shuffling was carried out with some amendments to the original protocol [15]. Two PCR purified products were mingled equally and fragmented by DNase I for 10 min. After purification, 50–100 bp fragments were utilized for primerless PCR reaction to reassemble into a full length with the conditions: 300 s denaturation at 95°C, then 30 cycles of thermal cycling (94°C for 30 s, 45°C for 45 s, 72°C for 120 s), 420 s of final extension at 72°C. Then, the reassembled gene of the exact size was obtained with 10-fold of diluted primerless reaction product as template, CotA-3F/CotA-3R as specific primers and PCR was performed as follows: 94°C for 60 s, 45 cycles of (30 s at 94°C, 30 s at 49°C, 30 s at 72°C), and a final extension at 72°C for 600 s. The purified PCR products digested by *Bam* HI and *Xho* I were ligated to a similarly digested pETDuet-1expression vector and the resulting recombinants were transformed into *E. coil* strains BL21 (DE3) for screening of evolved variants.

### Library screening and enzyme activity assay

Library screening was performed using high-throughput screening (HTS) method described as described previously with some modification [10]. In brief, single colonies of each engineered strain were cultivated into 200μL Luria-Bertani (LB) medium with 100μg/ml ampicillin in 96-well plates at 37°C overnight, 200rpm. Overnight culture (20μL) was inoculated into fresh 180 μL LB medium supplemented with the same antibiotic and cultivated at 37°C,200 rpm. At OD_600_ = 0.6, 0.1 mM IPTG and 0.25 mM CuSO_4_ were added. The culture was induced at 16°C. The shaker was switched off 4 h after induction, and the plates were kept in a stationary state at 16°C for overnight.

After induction, the cells were harvested by centrifugation (30 min, 3500 × g, 4°C). The precipitates were resuspended in BugBuster Protein Extraction Reagent. The cell extract was centrifuged at 3500 × g for 30 min, and the supernatant was saved as the cytoplasmic fraction.

Laccases activity was determined using ABTS and SGZ as substrates as previously described [16]. One unit is defined as the amount of enzyme that oxidizes 1 μmol of substrate per min at 30°C. All assays were performed in triplicate.

### Biochemical characterization

The CotA-laccase was purified from the highest enzymic activity mutant 5E29. Brief, the cytoplasmic fraction supernatant was filtered through a 0.45 μm filter to remove cell debris. it was loaded onto HisTrap^TM^ HP affinity column. The target protein was eluted by imidazole at different concentrations according to the protocol (GE healthcare, PA, USA). The elution fractions were concentrated and desalted by ultrafiltration (10 kDa cutoff, Milipore, Bedford, MA, USA).

Kinetic constants (K_m_, *k*_cat_ and *k*_cat_/K_m_) were determined using the Michaelis-Menten equation. K_m_ and *k*_cat_ values for mutants were compared with those of the wild-type CotA. The effect of pH on the activity of laccases was measured in a series of citrate-phosphate buffer with different pH (3.0–7.8, 0.1 M) and Tris – HCl buffer (7.4–9.0, 0.1 M) using ABTS and SGZ as substrate [17]. For pH stability assay, laccases for incubating in each pH (3.0–9.0) at 30°C for overnight, the remaining enzymatic activity was assayed with SGZ as substrate. The optimal temperature for the activity of laccases towards SGZ was determined at different temperatures ranging from 20°C to 90°C at optimal pH for 10 min. Thermal stability was determined at three elevated temperatures (60°C, 70°C, and 80°C) in 100 mM citrate-phosphate buffer (pH 6.8). The residual activity was measured at 30°C using SGZ as substrate.

### Dye decolorization

Indigo carmine (λ_max_ = 610 nm) and Congo red (λ_max_ = 475 nm) were used to evaluate the decolorization ability of wild-type and variant CotA-laccase. The reaction solution (6 mL) including 0.5 U/mL crude laccase, 25 mg/L indigo carmine or 80 mg/L Congo Red, 0.1 M Tris – HCl buffer (pH 9.0) and 0.1 mM acetosyringone was incubated at 37°C, 150 rpm. The decolorization efficiency was determined at different time intervals (1, 2, 4, 6, 8, 10 and 24 h) and analyzed for dye removal. All reactions were carried out in triplicate.

## Results and discussion

### Design, cloning, and expression of mutants

*E. coli* is the most frequently used host primarily owning to its capability of amenability to genetic manipulation [18]. In our research, five expression vectors were used, pET-20b(+), pET-22b(+), pET-30b(+), pET-39b(+) and pETDuet-1, to construct five engineered strains for screening a highest level of soluble protein. Low temperature and microaerobic conditions were used to improve the production of laccase in *E. coli* BL21 cells (DE3) [19]. Unfortunately, only the recombinant laccase constructed by the pETDuet-1 expression vector displayed color change using laccase substrates SGZ. This phenomenon is the same as described by earlier researchers, the recombinant laccases in *E. coli* often form inactive inclusion bodies [4,11].

**Figure 1.**
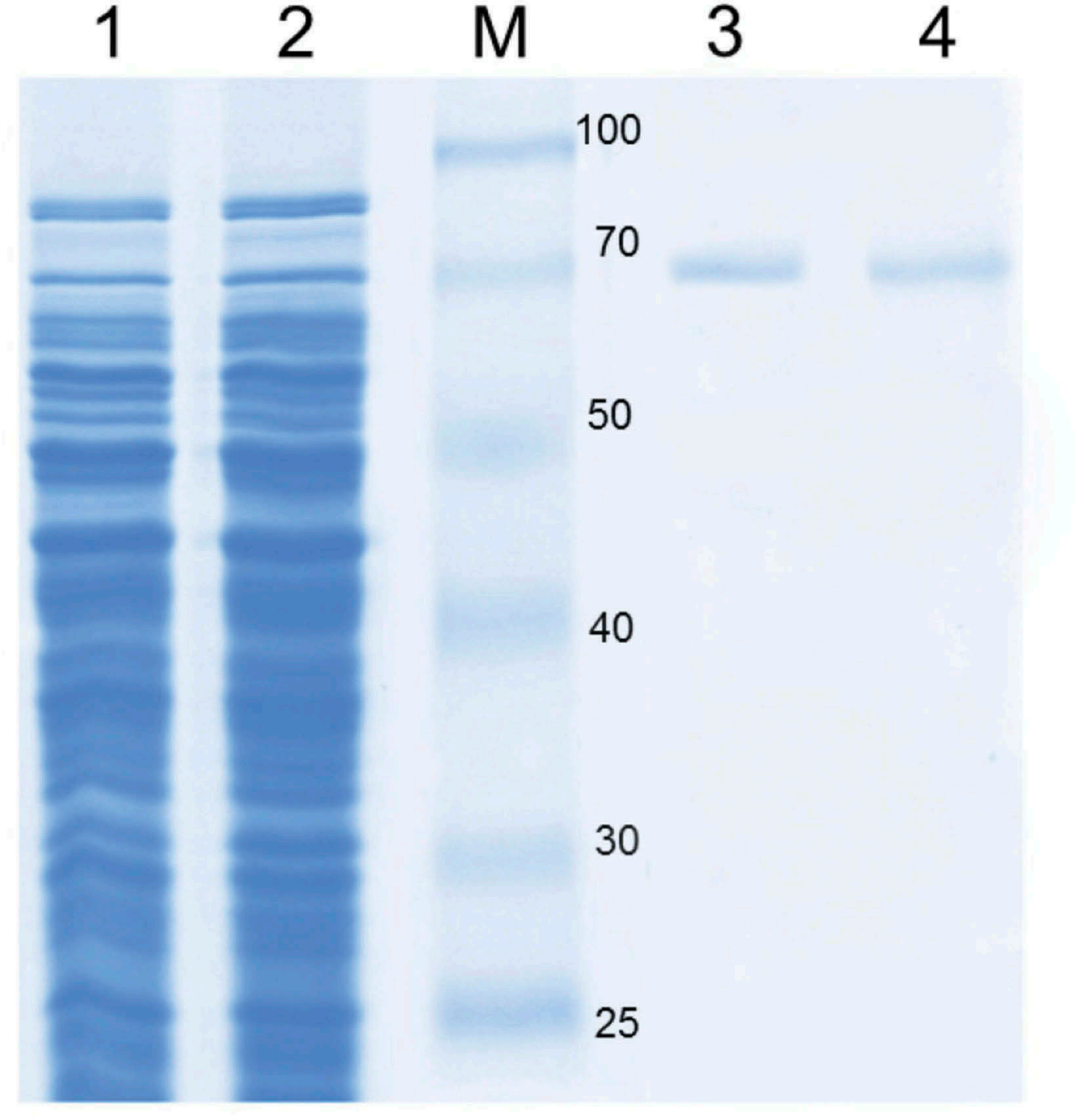
SDS-PAGE analysis of CotA-laccase. Protein samples were loaded in each lane as follows: cell extract wild-type CotA (lane 1), cell extract 5E29 CotA (lane 2), molecular weight marker (M), purified wild-type CotA (lane 3), purified 5E29 CotA (lane 4).

The open reading frame of *B. subtilis* LS03 and *B. amyloliquefaciens* LS05 CotA-laccase genes wascloned, and the sequence was deposited in the GenBank database under accession No.GU972588 and No.GU972590. Sequence analysis showed that different sequences of CotA-laccase genes were found in different *Bacillus* sp. strains. In this study, CotA-laccase genes from *B. subtilis* and *B. amyloliquefaciens* were both 1542 bp encoding a 512 amino acid protein. The CotA-laccase from *B. subtili*s had an amino acid identity of 77% with CotA-laccase from *B. amyloliquefaciens* (see Supplementary data Figure S1 for sequence information). Random mutations were introduced using DNA shuffling, and 4,000 recombinant clones were obtained. A mutation frequency of one to three mutations per gene was determined by sequencing 10 randomly selected clones. One-thousand six-hundred clones were analyzed crude enzyme activity towards the typical substrate SGZ to screen CotA-laccase variants. Forty-two percent clones had no activity or a little activity towards SGZ, and 56% clones analyzed had similar activity with the wild-type (0.86 U/mL) using SGZ as substrate. The case was expected, since most variants in DNA shuffling library possesses highly conserved functionally essential regions, which will be unable to tolerate amino acid changes to their protein 3D structures and will thus exhibit no or poor activity [20]. A single clone (5E29), which had a 4.45-fold higher activity (3.83 U/mL) than the wild-type, was screened. The sequence analysis of this variant revealed that its genes were not a sufficiently diverse recombined. This variant only possessed two missense mutations (T232P/Q367R), in comparison to the wild-type (supplementary Figure S2). The plasmids carrying the wild-type and 5E29 variant genes were transformed into *E.coli* BL21 (DE3) for expression. After a set of purification processes, the specific activity of mutant 5E29 reached 9.39 U/mg, with a 3.96-fold increase in comparison to that of the wild-type (2.37 U/mg). SDS-PAGE analysis of the wild-type enzyme and its variant showed a molecular weight of 65 kDa which was the same as that of other CotA-laccases (Figure 1.) [21].

### Characterization of 5E29 mutant

**Figure 4.**
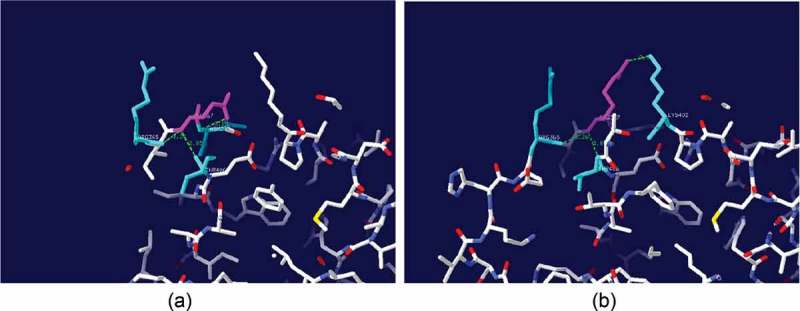
Local structure of the wild-type laccase and its variants. (a) The position of Gln367 and neighboring residues of the wild-type laccase (b) Gln-367-Arg.

Kinetic analysis of purified 5E29 mutant (T232P/Q367R) is shown in Table 2. Data were analyzed using plots of the initial oxidation rates vs. substrate concentration according to the Michaelis-Menten model (see Supplementary data Figure S3). Wild-type laccase showed *k_cat_* and K*_m_* values of 6.3 s^−1^ and 24.8 μM, respectively, using SGZ as the substrate. 5E29 mutant has elevated laccase catalytic efficiency through increased *k_cat_* and decreased K_m_ values. As shown in Figure 4(a), in the native form of the laccase, Gln-367 might be involved in hydrogen bonds with Arg-365, Asn-368, and Thr-406. Hence, replacing this residue with any of the amino acids will disrupt these connections. In the case of Arg substitution, a new bond between Arg-367 and Lys-402 can be formed (Figure 4(b)). In the case of Q367R, the binding pocket might have changed into a potentially more favorable binding site. The *k_cat_*/K_m_ value of 5E29 mutant was determined to be 0.374 s^−1^ µM^−1^ which achieved 1.47-fold higher catalytic efficiency (18.15% decrease in K_m_ and 1.21 fold increases in *k_cat_*) in relation to that of the wild-type. The *k_cat_* value was affected by the donor–acceptor electronic coupling, the reorganization energy and the redox potentials of the enzyme [22]. The 5E29 mutation might be due to increase of redox potential and feasibility of internal electron transfer rate. This single mutation of 5E29 seems to impose minimal effect on the binding and oxidative ability of a reducing substrate.

**Table 2.** Kinetic constants for the wild-type CotA and 5E29 mutant using SGZ as substrate.

Laccase	K_m_ (µM)	*k_cat_* (s^−1^)	*k_cat_*/K_m_ (s^−1^ µM^−1^)
WT	24.8 ± 3.4	6.3 ± 0.9	0.254
5E29	20.3 ± 1.3	7.6 ± 2.7	0.374

**Figure 2.**
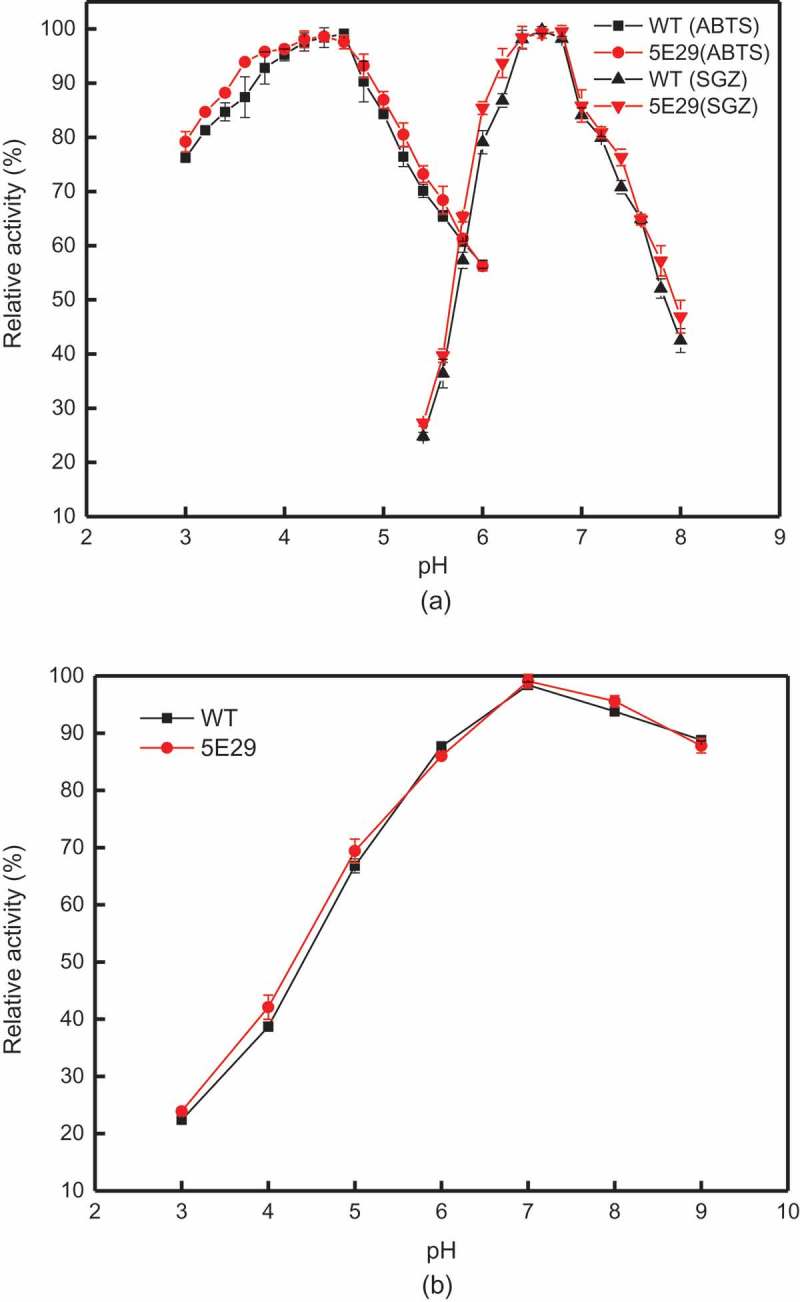
Effect of pH on the activity (a) optimal pH of wild-type CotA and 5E29 mutant for catalyzing ABTS and SGZ; (b) pH stability of wild-type CotA and 5E29 mutant.

The optimal pH values for the activity of 5E29 laccase towards ABTS and SGZ were 4.4 and 6.8, respectively (Figure 2(a)), while the pH optimum values of wild-type CotA for ABTS and SGZ were 4.6 and 6.6, respectively. Stability studies of pH showed 5E29 mutant is not suitable to work at pH 3.0 and lost 74% of its activity after 1-day. However, this variant is highly stable at pH 7.0–9.0, retaining approximately 85% of the initial activity after incubation for 24 h. The pH stability of 5E29 had no distinct difference with that of the wild-type (Figure 2(b)).

**Figure 3.**
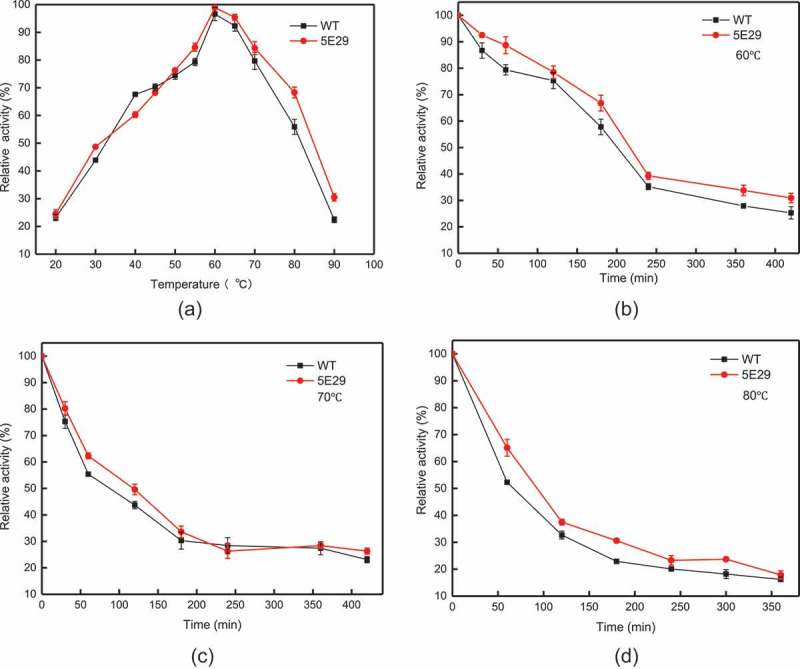
Effect of temperature on the activity (a) optimal temperature of wild-type CotA and 5E29 mutant for catalyzing SGZ; The thermostability of wild-type CotA and 5E29 mutant (b) at 60°C, (c) 70°C, (d) 80°C.

The optimal temperature for 5E29 mutant to oxidase SGZ was 60°C, which is the same as wild-type CotA (Figure 3(a)). High thermos stability is the most remarkable property of CotA-laccase [23]. As shown in Figure3(b–d), the thermal stability of 5E29 was a slight improvement compared to the parental laccase. This occurrence may have resulted from different the hydrophobic interactions of amino-acid residues. The half-life of wild-type CotA was 200 min, 86 min and 66 min at different temperatures (60°C, 70°C, and 80°C), and the half-life of inactivation of 5E29 was 216 min, 120 min and 90 min, respectively. The wild-type CotA retained 52.26% of its initial activity after 60 min incubation at 80°C, and 5E29 had 65.09% residual activity under the same conditions. As a result, 5E29 and wild-type CotA displayed excellent thermal stability. However, CotA from *B. licheniformis* demonstrated a weaker thermal stability which lost about 92% of its activity in the same condition [21].

The hydrophobic interactions of amino acid residues around the active center play an important role in determining thermal stability of enzymes [24]. Gln-367-Arg has imposed positive charge instead of the initial negative charge in this location and leads to formation of a new cation–π interaction. In addition, at position 232, a Thr-to-Phe substitution increased the hydrophobic interaction on the protein surface. Therefore, potential amino-acid substitutions had promoted the thermal stability of 5E29 mutant.

### Decolorization of dyes by 5E29 mutant

**Figure 5.**
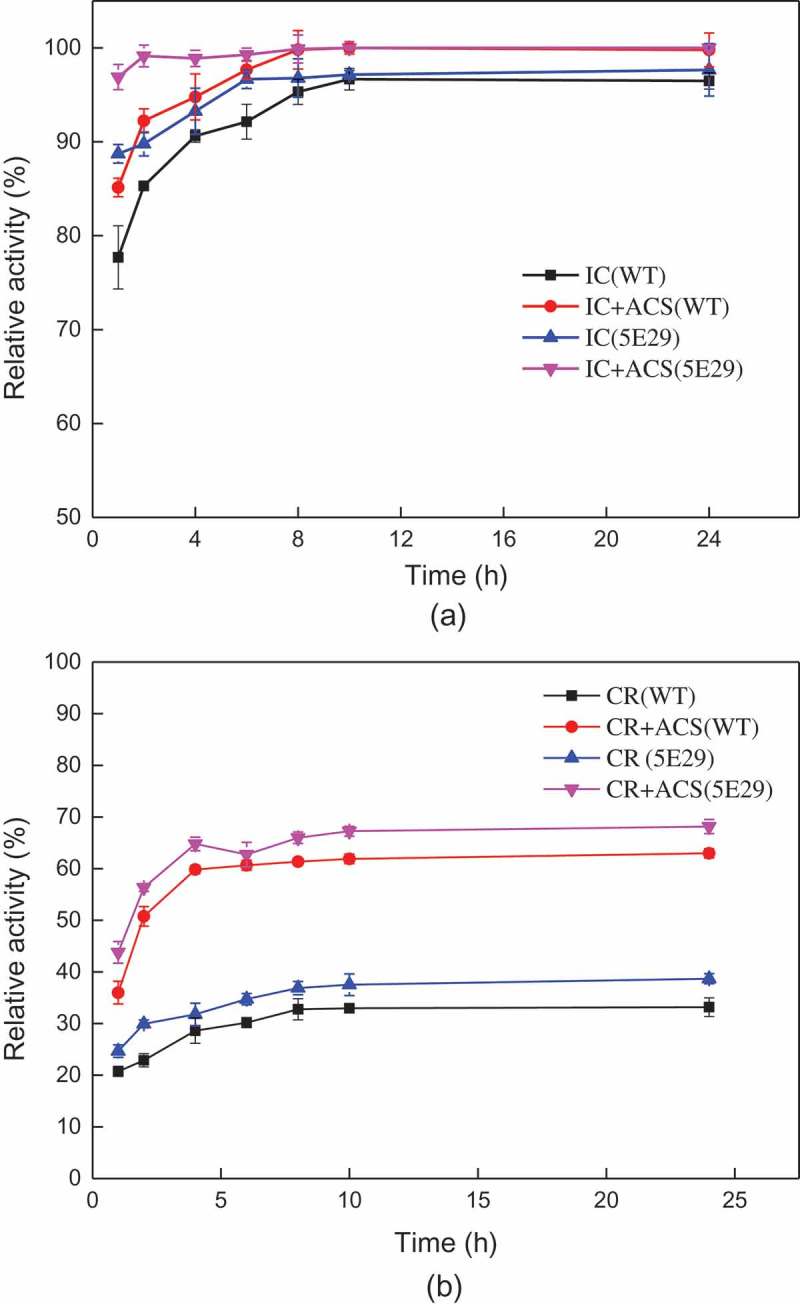
Decolorization of synthetic dyes by 5E29 and the wild-type CotA-laccases. (a) IC: Indigo carmine (b) CR: Congo Red.

The application of *B. subtilis* spores laccases for the decolorization of industrial dyes has been previously reported [25]. This study was evaluated the decolorization ability of the laccases using different industrial dyes with or without a redox mediator [9] (Figure 5). The decolorization rate of 5E29 was increased compared with the wild-type CotA. After incubation for 2 h, the decolorization efficiency of Indigo Carmine reached 92.2% without a redox mediator. However, the maximum decolorization rate of Congo Red was only approximately 34.7% without a mediator after 6 h of incubation. Acetosyringone is a laccase mediator which cannot cause secondary pollution of the environment. The addition of 0.1 mM Acetosyringone could further improve the decolorization efficiency for dyes. Approximately 52.7% decolorization was observed for Congo Red after 6 h with adding 0.1 mM Acetosyringone. Hence, a laccase-mediated system could provide a feasible solution to the decolorization of azo dyes. It was assumed that the enhanced decolorization ability may have resulted from the changes in electrostatic interactions between the redox mediator and the catalytic residues after mutation occurred [9].

## Conclusion

Heterologous expression of CotA laccases in *E. coli* exhibited low catalytic efficiency was unsatisfactory to meet industrial demands. In this study, we focused on random mutagenesis by DNA shuffling in CotA-laccase. A T232P/Q367R-CotA variant with increased catalytic activity was constructed. This mutant with Acetosyringone was highly effective in the degradation of azo dyes under alkaline condition.
