# Supplementary material for: Enhanced catalytic efficiency of CotA-laccase by DNA shuffling
Source: Bioengineered. 2019 May 29;10(1):182–9. doi: 10.1080/21655979.2019.1621134 (PMC6550543; doi:10.1080/21655979.2019.1621134)

Journal name: Bioengineered

Supplementary data

Manuscript title: Enhancement the catalytic efficiency of a CotA-laccase from *Bacillus subtilis* by a DNA shuffling strategy

Fengju Ouyang^a^ and Min Zhao^b^*

^a^ Institute of advanced technology, Heilongjiang Academy of science, Harbin, China; ^b^* Northeast Forestry University, Department of Microbiology. Harbin, China

**Corresponding author:** Min Zhao

**Mailing address:** Address: No.26, Hexing road, Xiang fang district, Harbin, Heilongjiang, 150040, CN

**Tel.:** +86 0451 82191513;

**Fax:** +86 0451 82191513

**E-mail:** 82191513@163.com

**Fig. S1 Amino acid sequence alignment of parent mutants( LS03 and LS05).** The open reading frame of *B. subtilis* LS03 and *B. amyloliquefaciens* LS05 cotA genes sequence were deposited in the GenBank database under accession No.GU972588 and No.GU972590. Sequence analysis showed that different gene sequences of cotA were found in different strains of the same Bacillus species. In this study, cotA from *B. subtilis* and *B. amyloliquefaciens* which were both 1542 bp encoding a 512 amino acid protein. The CotA laccase from *B. subtilis* LS03 had an amino acid identity of 77% with laccase from *B. amyloliquefaciens* LS05.


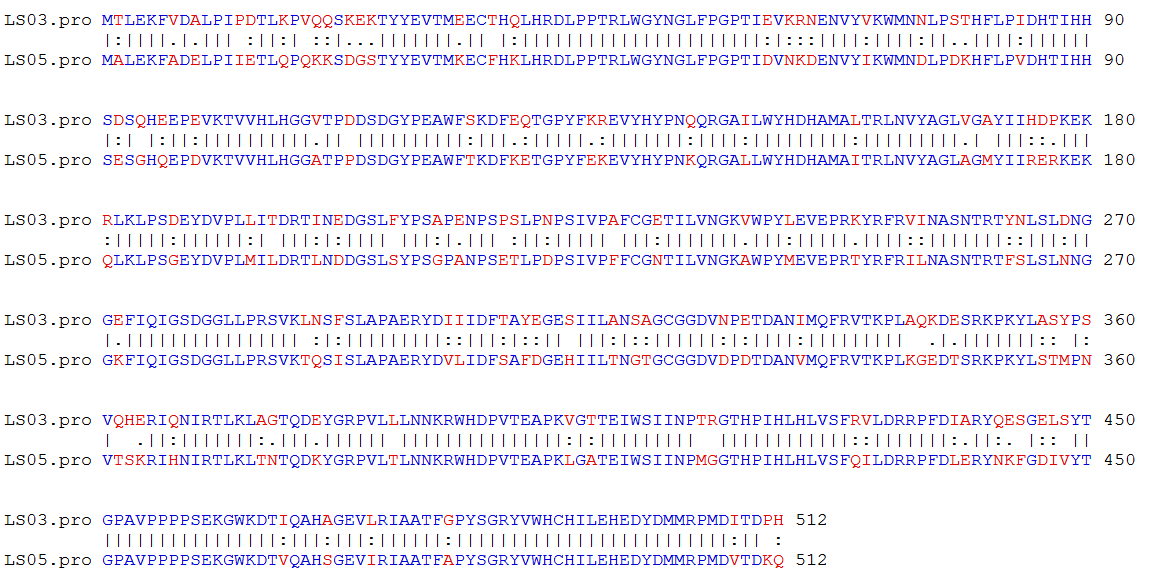


**Fig. S2 pETDuet-1 Vector. pETDuet-1 is as the expression vector in this study.** The vector contains two multiple cloning sites (MCS), each of which is preceded by a T7 operator and a ribosome binding site (rbs). The vector also carries the pBR322-derived ColE1 replicon, *lacI* gene ampicillin resistance gene. The S·Tag coding sequence make the recombinant purification easier.


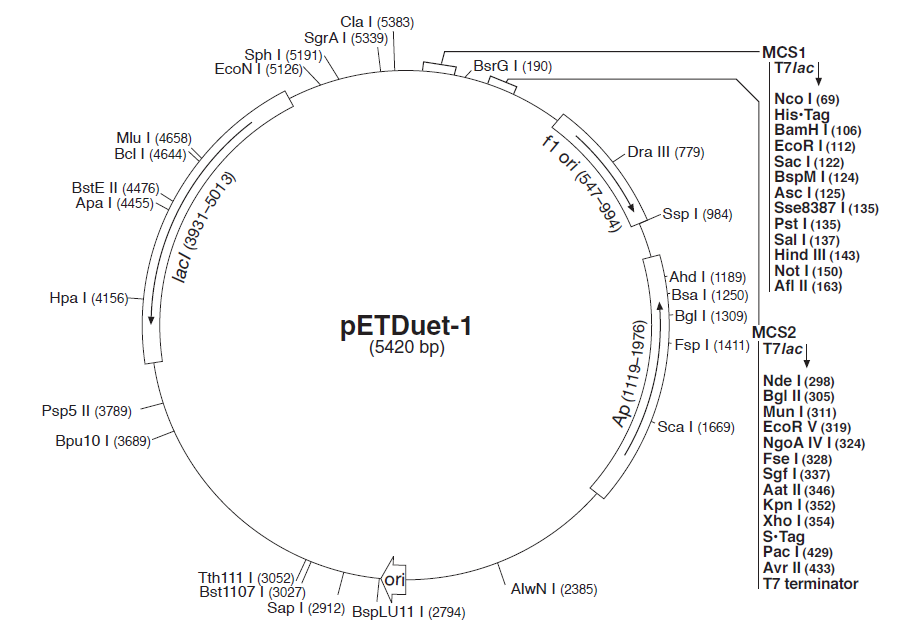


**Fig. S3. Dates analyzed using plots of the initial oxidation rates vs. substrate concentration according to the Michaelis-Menten model. (A) wild-type CotA, (B)** **5E29 CotA-laccase.**


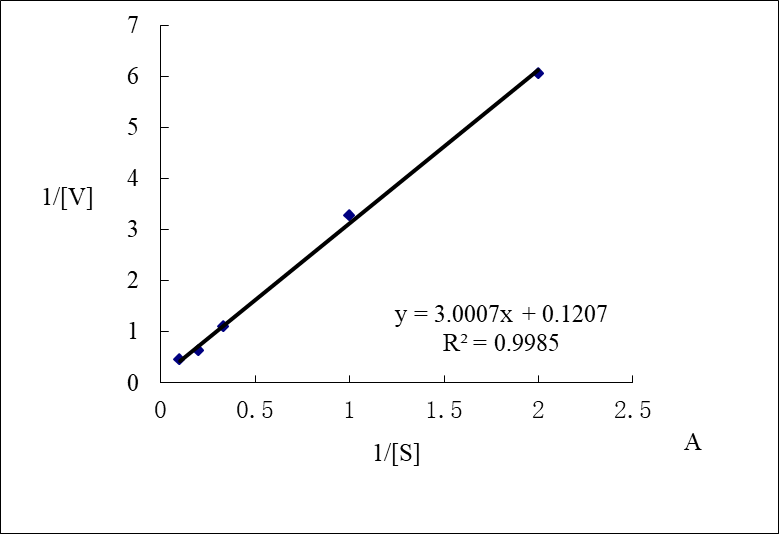

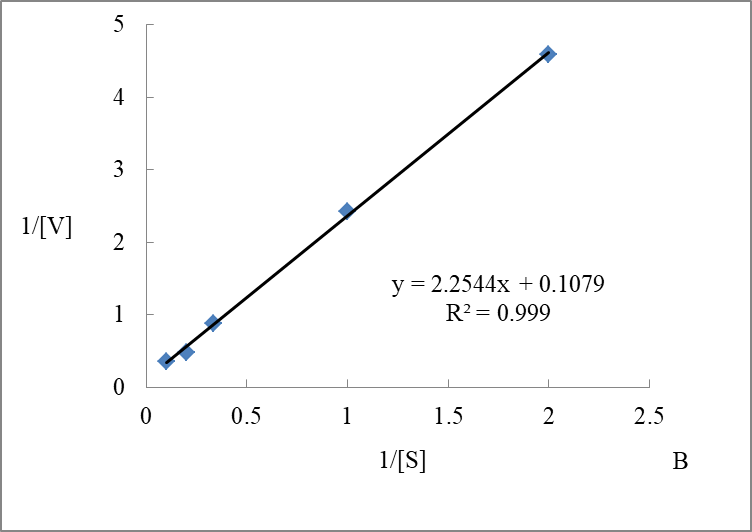

Supplement: Supplemental Material [file kbie-10-01-1621134-s001.docx]
